# Supplementary material for: Post-translational toxin modification by lactate controls Staphylococcus aureus virulence
Source: Nat Commun. 2024 Nov 13;15:9835. doi: 10.1038/s41467-024-53979-8 (PMC11561239; doi:10.1038/s41467-024-53979-8)
Supplement: Supplementary file 1 — Supplementary Information [file 41467_2024_53979_MOESM1_ESM.pdf]

Post-translational toxin modification by lactate controls *Staphylococcus aureus* virulence

Yanan Wang, Yanfeng Liu, Guoxiu Xiang, Ying Jian, Ziyu Yang, Tianchi Chen, Xiaowei Ma, Na Zhao, Yingxin Dai, Yan Lv, Hua Wang, Lei He, Bisheng Shi, Qian Liu, Yao Liu, Michael Otto, Min Li

Supplementary Information

This Supplementary Information file contains:

Supplementary Figures 1-5
Supplementary Tables 1-3

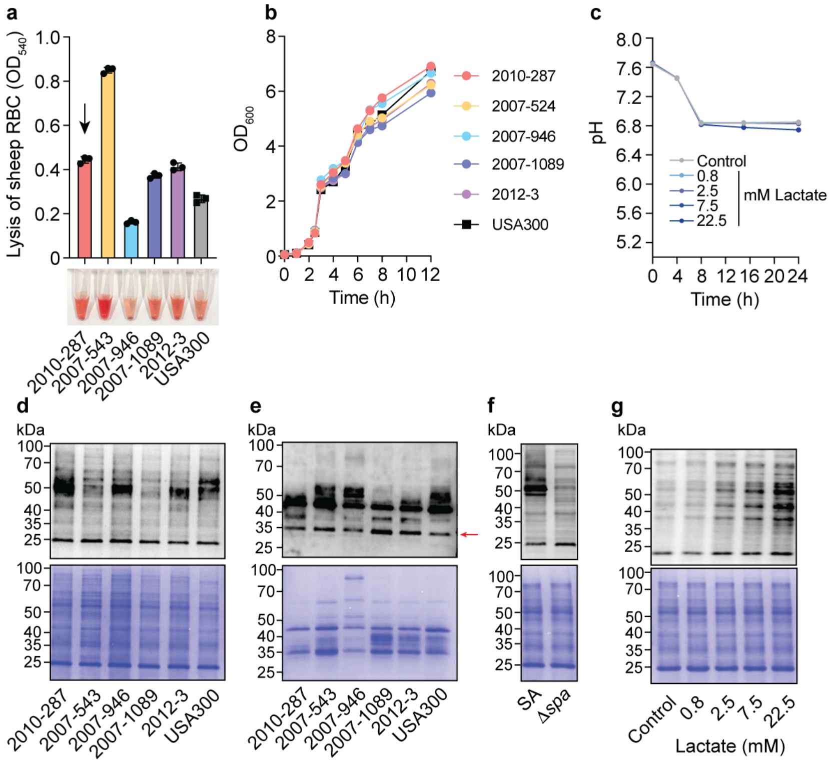

Supplementary Figure 1. Strain selection and lactylation of cytoplasmic proteins. (a), Cytolytic capacity toward RBCs of culture filtrates from different ST398 strains and strain USA300. The black arrow shows the selected strain. n=3/group (biological replicates). **(b)**, Growth curves of the same strains. **(c)**, pH of *S. aureus* cultures with different additions of

lactate. n=3/group (biological replicates). **(d,e)**, Lactylated proteins determined using immunoblot (Pan-αKla antibody) in the cellular fraction (**d**) and culture filtrates (**e**). Alpha-toxin is marked by a red arrow. **(f)**, Lactylation of proteins in the cellular fraction of *S. aureus* and *S. aureus* Δ*spa*. **(g)**, Lactylation of *S. aureus* Δ*spa* proteins in the cellular fraction with addition of different levels of sodium lactate to cultures. **(d-g)**, Coomassie total proteins stains are shown at the bottom as loading controls. **(f,g)**, Conditions are the same as those used for the experiments shown in the main **Fig. 1e,f**.

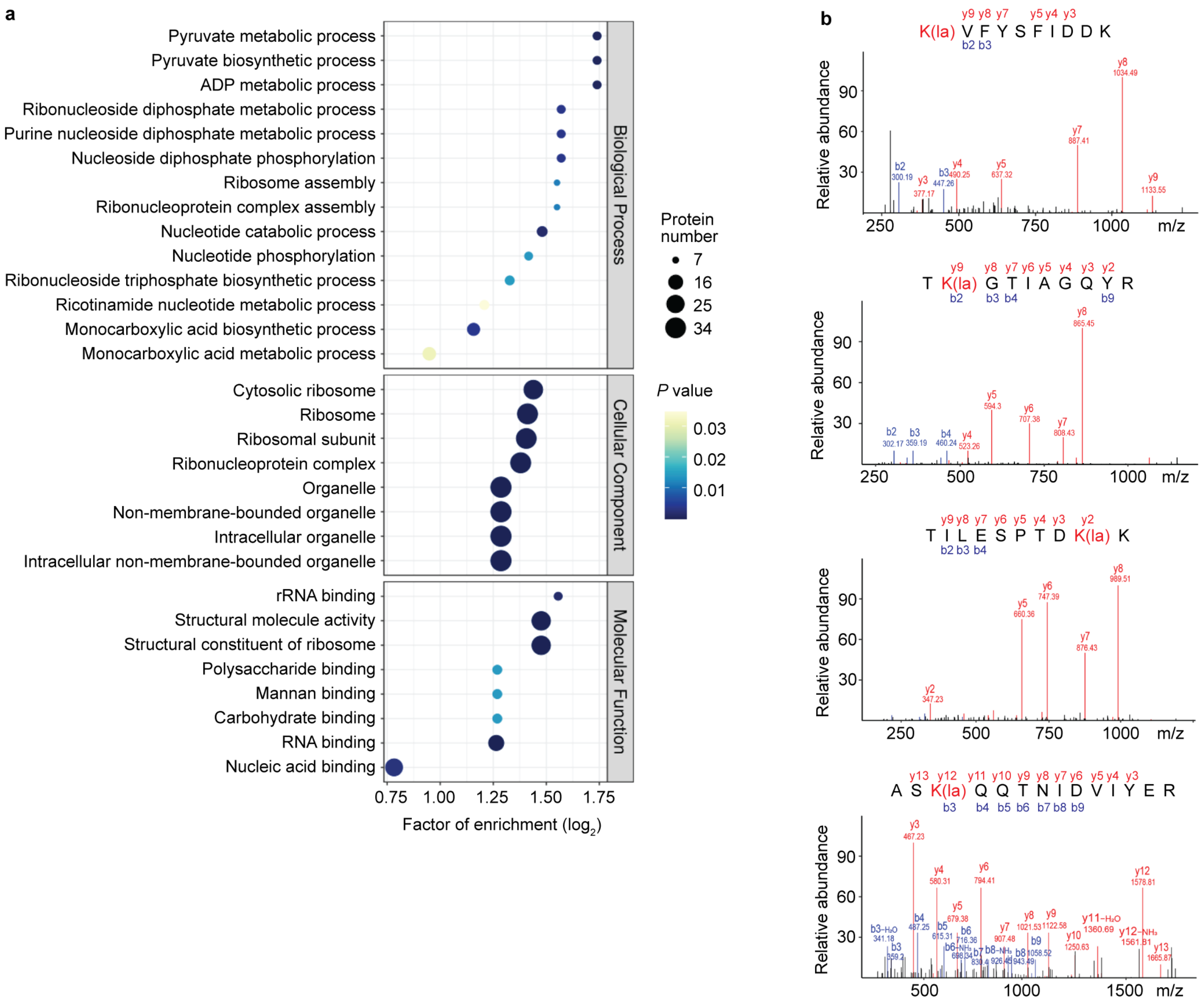

Supplementary Figure 2. LC-MS/MS analysis of protein lactylation. (a), Lactylated cytoplasmic proteins according to LC-MS/MS analysis, Gene ontology (GO) annotation analysis. Statistical analysis is by two-sided Fisher's exact test versus all identified proteins or groups. **(b)**, Identified lactylated peptides in alpha-toxin.

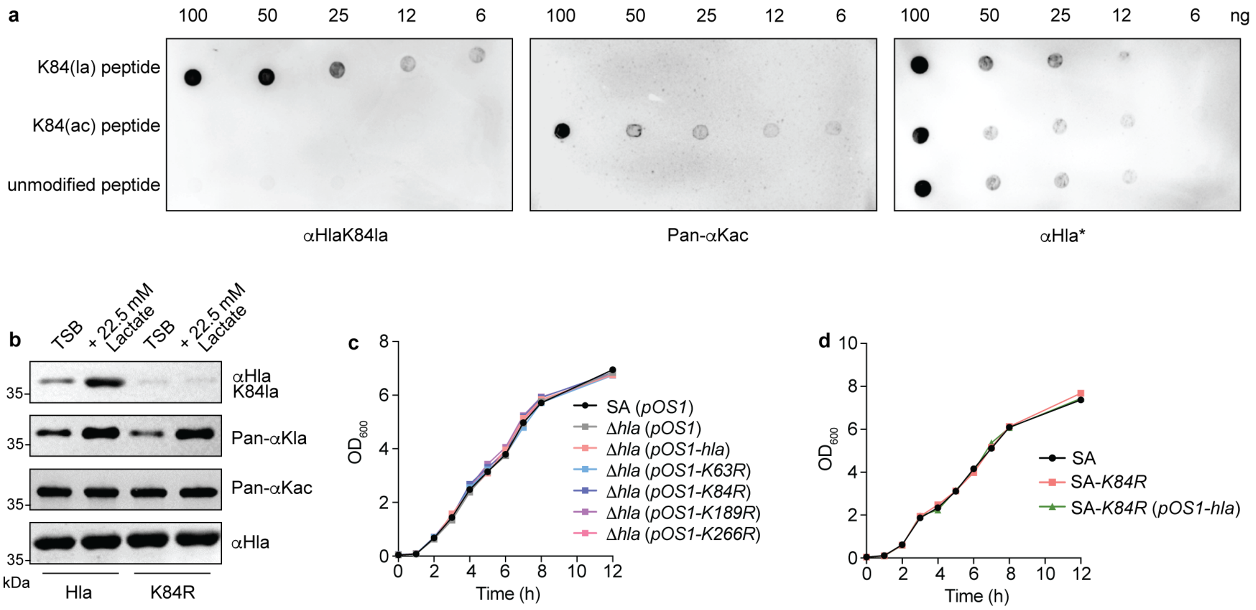

Supplementary Figure 3. Background information for site-specific lactylation experiments. **(a)**, Test of specificity of the developed K84la-specific antibody (αHlaK84la). Antibodies specific for alpha-toxin (αHla*, developed against the unmodified K84 region peptide VIRTKGTIAG), K84-lactylated alpha-toxin (αHlaK84la, developed against the corresponding K84-lactylated peptide), and antibodies reacting with all lysine-acetylated proteins (Pan-αKac) were tested for reaction with unmodified, lactylated, and acetylated peptide (see main **Figure 3c** for peptide sequence). **(b)**, Reaction of equal amounts of alpha-toxin (wild-type and K84R derivative) with αHlaK84la, Pan-αKac, Pan-αKla, and αHla antibodies, purified from cultures with and without addition of sodium lactate. **(c)**, Growth curves of strains expressing different single-site amino acid substitutions. **(d)**, Growth curves of SA-*K84R* and *hla*-complemented strains in comparison to wild-type strain.

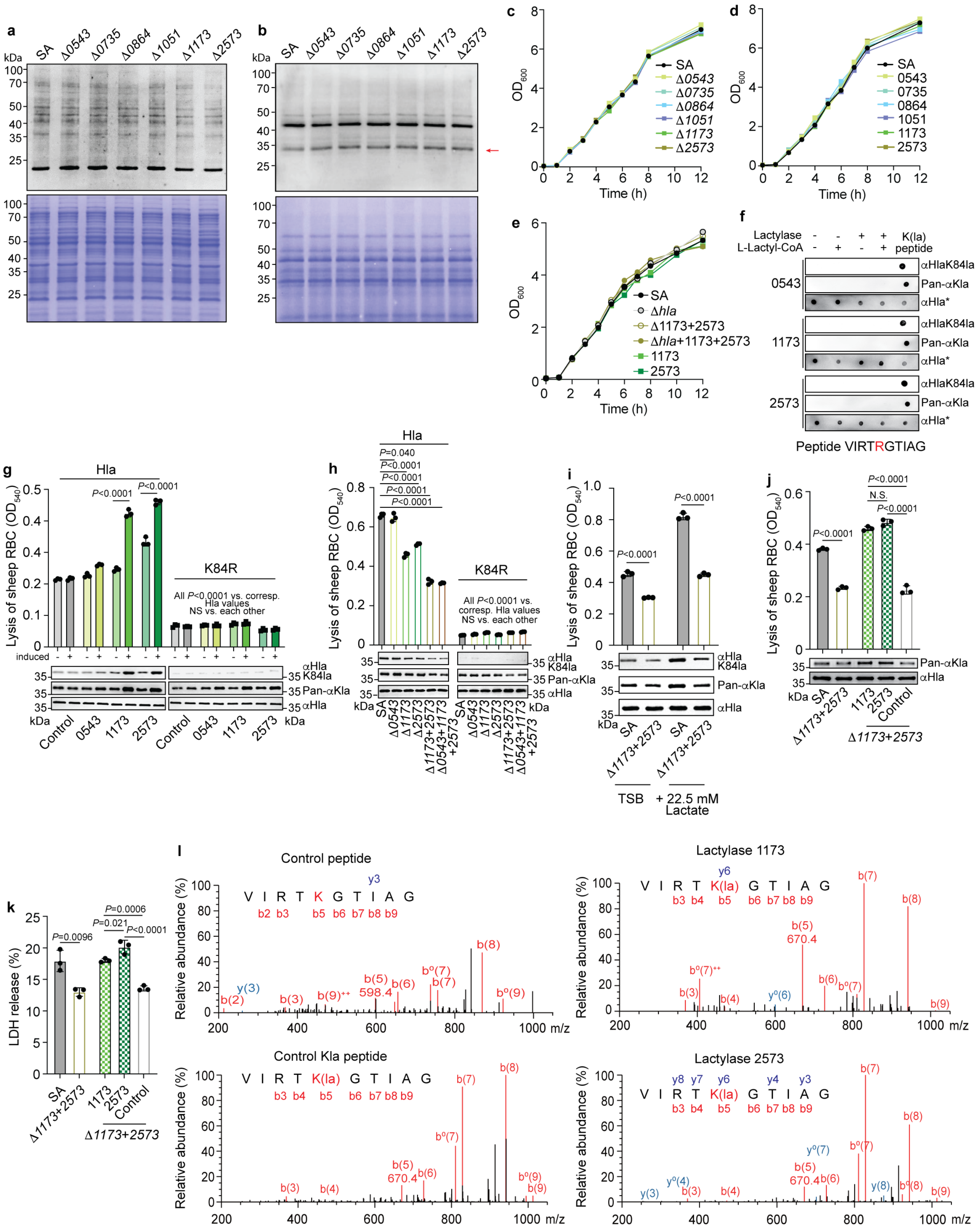

Supplementary Figure 4. Background information for lactylase deletion experiments. (a,b), Lactylated proteins determined using immunoblot (Pan-αKla antibody) in the cellular fraction (**a**) and culture filtrates (**b**) of constructed deletion strains. Alpha-toxin is marked by a red arrow.

Coomassie total proteins stains are shown at the bottom as loading controls. **(c-e)**, Growth curves of putative lactylase deletion (**c**), over-expression (**d**), and genetically complemented and control **(e)** strains. **(f)**, In vitro lactylase activity assay (K84R peptide controls). Purified recombinant lactylases were incubated with a K to R derivative peptide representing the region surrounding position 84 of Hla (see bottom for peptide sequence). Analysis was by immune dot blot using antibodies as described in the legend to **Figure 3**. **(g-k)**, Experiments using Hla purified from the indicated strains (rather than culture filtrates as in the corresponding experiments shown in **Figure 3**) n=3/group; biological replicates for all experiments. **(g)**, cytolysis of RBCs by lactylase expression strains. **(h)**, Cytolysis of RBCs in lactylase deletion strains. **(i)**, Lactate dependence of RBC cytolysis. **(j,k)**, Genetic complementation experiments showing RBC **(j)** and A549 cell **(k)** cytolysis. **(l)** Representative MS spectra related to the experiment shown in **Figure 3l**.

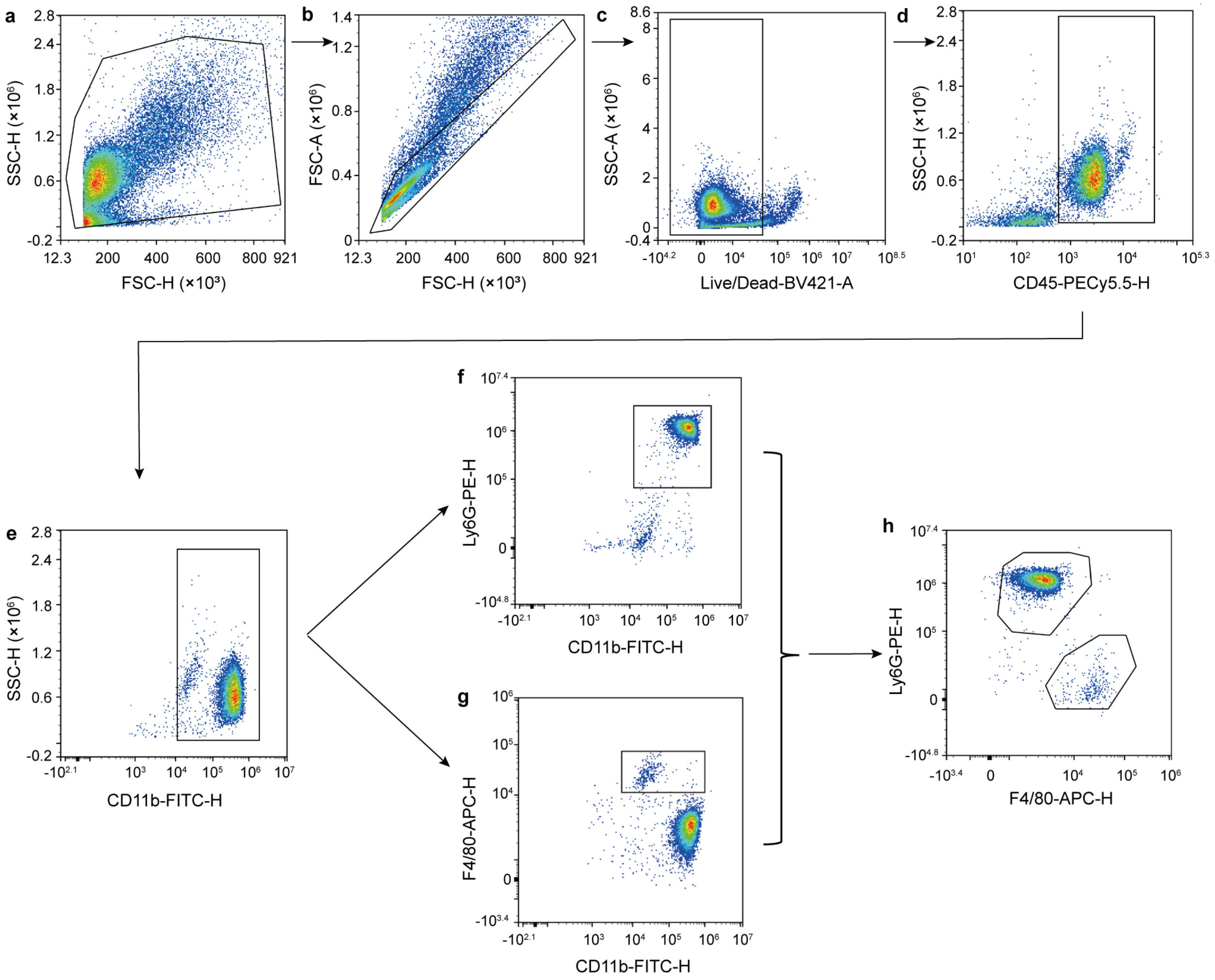

Supplementary Figure 5. Gating strategy to quantitate leukocyte populations in bronchoalveolar lavage fluid. BALF cells were examined initially by forward scatter (FSC) height versus side scatter (SSC) height (**a**), and FSC height versus FSC area (**b**), with gating on single cells to eliminate debris and clumped cells from the analysis. Subsequently, a Live/Dead dye was used to eliminate dead cells (**c**). Live cells were then examined by CD45 expression, gating on $CD45^+$ cells, which represented total leukocytes (**d**). Live $CD45^+$ cells were then examined based on CD11b expression, gating on $CD11b^+$ cells, which represented myeloid cells (**e**). Then, neutrophils were defined as $CD45^+CD11b^+$ $Ly6G^+$ cells (**f**), and macrophages were defined as $CD45^+CD11b^+$ $F4/80^+$ cells (**g**). Examination of these $CD11b^+$ cells by Ly6G versus F4/80 expression allows the discrimination of two cell populations: $Ly6G^+$ neutrophils and $F4/80^+$ macrophages (**h**).

Supplementary Table 1. Bacterial strains and plasmids used in this study.

Strains/plasmids	Relevant genotype and property	Source/reference
E. coli		
DH5α	*endA1 recA1 gyrA96 thi-1 hsdR17 (rK-mK+) relA1 supE44 (lacZYA-argF) U169 F-80dlacZM15 deoR phoA*	Invitrogen
BL21 (DE3)	Expression strain, *F-*, *ompT*, *hsdS (rBB-mB —), gal, dcm(DE3)*	Invitrogen
S. aureus		
RN4220	derived from NCTC8325-4; r-m+	[1]
USA300	USA300 LAC, sequence type 8 (ST8)	[2]
ST398	clinical isolate	This study
Δ*spa*	*spa* deletion mutant of ST398	This study
Δ*hla*	*hla* deletion mutant of ST398	This study
ST398 *(pOS1)*	ST398 carrying plasmid *pOS1*	This study
Δ*hla (pOS1)*	Δ*hla* carrying plasmid *pOS1*	This study
Δ*hla (pOS1-hla)*	Δ*hla* carrying plasmid *pOS1-hla* with His-tag	This study
Δ*hla (pOS1-K63R)*	Δ*hla* carrying plasmid *pOS1-hla (K63R* mutant*)* with His-tag	This study
Δ*hla (pOS1-K84R)*	Δ*hla* carrying plasmid *pOS1-hla (K84R* mutant*)* with His-tag	This study
Δ*hla (pOS1-K189R)*	Δ*hla* carrying plasmid *pOS1-hla (K189R* mutant*)* with His-tag	This study
Δ*hla (pOS1-K266R)*	Δ*hla* carrying plasmid *pOS1-hla (K266R* mutant*)* with His-tag	This study
SA-*K84R*	*hla (K84R)* genomic mutant of ST398	This study
SA-*K84R* (*pOS1-hla*)	*hla (K84R)* genomic mutant of ST398 carrying plasmid *pOS1-hla* with His-tag	This study
Δ*spa*Δ*0543*	*spa* and *0543* double-deletion mutant of ST398	This study
Δ*spa*Δ*0735*	*spa* and *0735* double-deletion mutant of ST398	This study
Δ*spa*Δ*0864*	*spa* and *0864* double-deletion mutant of ST398	This study
Δ*spa*Δ*1051*	*spa* and *1051* double-deletion mutant of ST398	This study
Δ*spa*Δ*1173*	*spa* and *1173* double-deletion mutant of ST398	This study
Δ*spa*Δ*2573*	*spa* and *2573* double-deletion mutant of ST398	This study
Δ*0543*	*0543* deletion mutant of ST398	This study
Δ*1173*	*1173* deletion mutant of ST398	This study
Δ*2573*	*2573* deletion mutant of ST398	This study

Δ*1173+2573*	*1173* and *2573* gene deletion mutant of ST398	This study
Δ*0543+1173+2573*	*0543*, *1173* and *2573* gene deletion mutant of ST398	This study
Δ*1173+2573* (*pYJ335-1173*)	*1173* and *2573* gene deletion mutant of ST398 carrying plasmid *pYJ335-1173*	This study
Δ*1173+2573* (*pYJ335-2573*)	*1173* and *2573* gene deletion mutant of ST398 carrying plasmid *pYJ335-2573*	This study
Δ*1173+2573* (*pYJ335*)	*1173* and *2573* gene deletion mutant of ST398 carrying plasmid *pYJ335*	This study
Δ*hla*Δ*1173+2573* (*pYJ335-1173*, *pOS1-hla*)	*hla*, *1173* and *2573* gene deletion mutant of ST398 carrying plasmid *pOS1-hla* with His-tag and plasmid *pYJ335-1173*	This study
Δ*hla*Δ*1173+2573* (*pYJ335-2573*, *pOS1-hla*)	*hla*, *1173* and *2573* gene deletion mutant of ST398 carrying plasmid *pOS1-hla* with His-tag and plasmid *pYJ335-2573*	This study
Δ*hla*Δ*1173+2573* (*pYJ335*, *pOS1-hla*)	*hla*, *1173* and *2573* gene deletion mutant of ST398 carrying plasmid *pOS1-hla* with His-tag and plasmid *pYJ335*	This study
Δ*hla*Δ*0543* (*pOS1-hla*)	*hla* and *0543* gene deletion mutant of ST398 carrying plasmid *pOS1-hla* with His-tag	This study
Δ*hla*Δ*1173* (*pOS1-hla*)	*hla* and *1173* gene deletion mutant of ST398 carrying plasmid *pOS1-hla* with His-tag	This study
Δ*hla*Δ*2573* (*pOS1-hla*)	*hla* and *2573* gene deletion mutant of ST398 carrying plasmid *pOS1-hla* with His-tag	This study
Δ*hla*Δ*1173+2573* (*pOS1-hla*)	*hla*, *1173* and *2573* gene deletion mutant of ST398 carrying plasmid *pOS1-hla* with His-tag	This study
Δ*hla*Δ*0543+1173+2573* (*pOS1-hla*)	*hla*, *0543*, *1173* and *2573* gene deletion mutant of ST398 carrying plasmid *pOS1-hla* with His-tag	This study
Δ*hla*Δ*0543* (*pOS1-K84R*)	*hla* and *0543* gene deletion mutant of ST398 carrying plasmid *pOS1-K84R* with His-tag	This study
Δ*hla*Δ*1173* (*pOS1-K84R*)	*hla* and *1173* gene deletion mutant of ST398 carrying plasmid *pOS1-K84R* with His-tag	This study
Δ*hla*Δ*2573* (*pOS1-K84R*)	*hla* and *2573* gene deletion mutant of ST398 carrying plasmid *pOS1-K84R* with His-tag	This study
Δ*hla*Δ*1173+2573* (*pOS1-K84R*)	*hla*, *1173* and *2573* gene deletion mutant of ST398 carrying plasmid *pOS1-K84R* with His-tag	This study

Δ*hla*Δ*0543+1173+2573* (*pOS1-K84R*)	*hla*, *0543*, *1173* and *2573* gene deletion mutant of ST398 carrying plasmid *pOS1-K84R* with His-tag	This study
Δ*spa* (*pYJ335*)	Δ*spa* carrying plasmid *pYJ335*	This study
Δ*spa* (*pYJ335-0543*)	Δ*spa* carrying plasmid *pYJ335-0543*	This study
Δ*spa* (*pYJ335-0735*)	Δ*spa* carrying plasmid *pYJ335-0735*	This study
Δ*spa* (*pYJ335-0864*)	Δ*spa* carrying plasmid *pYJ335-0864*	This study
Δ*spa* (*pYJ335-1051*)	Δ*spa* carrying plasmid *pYJ335-1051*	This study
Δ*spa* (*pYJ335-1173*)	Δ*spa* carrying plasmid *pYJ335-1173*	This study
Δ*spa* (*pYJ335-2573*)	Δ*spa* carrying plasmid *pYJ335-2573*	This study
ST398-(*pYJ335*)	ST398 carrying plasmid *pYJ335*	This study
ST398-(*pYJ335-0543*)	ST398 carrying plasmid *pYJ335-0543*	This study
ST398-(*pYJ335-1173*)	ST398 carrying plasmid *pYJ335-1173*	This study
ST398-(*pYJ335-2573*)	ST398 carrying plasmid *pYJ335-2573*	This study
Δ*hla* (*pYJ335*, *pOS1-hla*)	Δ*hla* carrying plasmid *pYJ335* and *pOS1-hla* with His-tag	This study
Δ*hla* (*pYJ335-0543*, *pOS1-hla*)	Δ*hla* carrying plasmid *pYJ335-0543* and *pOS1-hla* with His-tag	This study
Δ*hla* (*pYJ335-1173*, *pOS1-hla*)	Δ*hla* carrying plasmid *pYJ335-1173* and *pOS1-hla* with His-tag	This study
Δ*hla* (*pYJ335-2573*, *pOS1-hla*)	Δ*hla* carrying plasmid *pYJ335-2573* and *pOS1-hla* with His-tag	This study
Δ*hla* (*pYJ335*, *pOS1-K84R*)	Δ*hla* carrying plasmid *pYJ335* and *pOS1-K84R* with His-tag	This study
Δ*hla* (*pYJ335-0543*, *pOS1-K84R*)	Δ*hla* carrying plasmid *pYJ335-0543* and *pOS1-K84R* with His-tag	This study
Δ*hla* (*pYJ335-1173*, *pOS1-K84R*)	Δ*hla* carrying plasmid *pYJ335-1173* and *pOS1-K84R* with His-tag	This study
Δ*hla* (*pYJ335-2573*, *pOS1-K84R*)	Δ*hla* carrying plasmid *pYJ335-2573* and *pOS1-K84R* with His-tag	This study
**Plasmids**		
pKOR1	*cmR* and *ampR*, temperature sensitive vector for allelic replacement via lambda recombination and *ccdB* selection	[3]
pOS1	*E. coli*/*Staphylococcus* shuttle cloning plasmid, *cmR*, *ampR*	[4]
pYJ335	*E. coli*/*Staphylococcus* shuttle cloning plasmid, *ermR*, *ampR*	[5]
pET28a	Expression vector with His-tag, *kanR*	Novagen

Supplementary Table 2. Oligonucleotides used in this study.

Oligonucleotide	Sequence
**Oligonucleotides for isogenic deletion mutants**	

hla-att1	TTATCCACTTCCAATGTTTTCGTTTTACCATTCCTCAAA
hla-rev1	TTTCATCATCCTTCTATTTTTTAAAACG
hla-rev2	CGTTTTAAAAAATAGAAGGATGATGAAATGGTTATGTACACTCAAATAGTCACA
hla-att2	TACTTCCAATCCAATGAATGACGAAGAAGTCCAAACAAA
spa-att1	TTATCCACTTCCAATGAAGTCAAGCCTGAAGTCGATATG
spa-rev1	ATTAATACCCCCTGTATGTATTTG
spa-rev2	CAAATACATACAGGGGGTATTAATAAACAAACAATACACAACGATAGAT
spa-att2	TACTTCCAATCCAATGTAAAATTCAAAAACGAACGCCTA
0543-att1	TTATCCACTTCCAATGTCGGCTTAACCAACATGTGA
0543-rev1	ATGTCCTCCGTAGGCATTTGA
0543-rev2	TCAAATGCCTACGGAGGACATTTGTTCATTTAAGAAGTAAAGG
0543-att2	TACTTCCAATCCAATGCATGAACGCTACCCGTAACA
0735-att1	TTATCCACTTCCAATGGCGGTCGCACCTTATTCTTA
0735-rev1	AAAAAGCCTCCAGTATTTTGAA
0735-rev2	TTCAAAATACTGGAGGCTTTTTAGCTATTTTATCATAATCTTGTA
0735-att2	TACTTCCAATCCAATGGCGGTTCTTTATCATTTCTGC
0864-att1	TTATCCACTTCCAATGGCTCAAAGATGGGAGCTCTT
0864-rev1	CTTTGCCCCTCCTTTTAGTTCTAT
0864-rev2	ATAGAACTAAAAGGAGGGGCAAAGTTTCAAGTTAGGATTACATT
0864-att2	TACTTCCAATCCAATGGCAGCCGCTAAACTAAATGC
1051-att1	TTATCCACTTCCAATGCATCCAAGTGTAGACAGCATTCA
1051-rev1	AAAATTCCTCCATATCCTAAT
1051-rev2	ATTAGGATATGGAGGAATTTTAAAAAGTTGTATCTATTATAG
1051-att2	TACTTCCAATCCAATGGCGGCTTTATATTGCGTTTC
1173-att1	TTATCCACTTCCAATGGTCGTCTCTGGCATCTCCATT
1173-rev1	ATTTATCTACCCCTTATTTG
1173-rev2	CAAATAAGGGGTAGATAAATGATAATAATATTCGACACTAC
1173-att2	TACTTCCAATCCAATGTCGTTGCTAACGGTGTAGGTT
2573-att1	TTATCCACTTCCAATGCGGTCCATTCTTCCAAGGTA
2573-rev1	CTCCTATCATGATTGATTATAGTA
2573-rev2	TACTATAATCAATCATGATAGGAGATAAACCTCGCGATGGTGCTCCA
2573-att2	TACTTCCAATCCAATGCCTTCTGGTGCCGTAAATGT

**Oligonucleotides for *hla* gene K84R mutation in the ST398 genome**	
K84R-att1	TTATCCACTTCCAATGTGATACGGCACCCCTGAATT
K84R -rev1	ACCTCTCGTTCTGATGACTAATATTTT
K84R -rev2	ATATTAGTCATCAGAACGAGAGGTACCATTGCTGGT CAGTATAGAG
K84R -att2	TACTTCCAATCCAATGAATAGCCGACGCACATCATT
**Oligonucleotides for genetic complementation**	
hla (with *hla* promoter)-*pos1*-EcoRI-F	GAGGAATTCATAATTAATACCCTTTTTCTC
hla-pos1-BamhI-his-R	GAGGGATCCTTAATGATGATGATGATGATGATTTGTC ATTTCTTCTTTTTCC
0543-pyj335-EcoRV-F	GAGGATATCATGCAAATATATTTAAGTACTT
0543-pyj335-KPNI-flag-R	GAGGGTACCTTACTTATCGTCGTCATCCTTGTAATCA TAAAAATGTTCTGGAAATTTA
0735-pyj335-EcoRV-F	GAGGATATCATGGCCCATATTATACGTAGA
0735-pyj335-KPNI-flag-R	GAGGGTACCTTACTTATCGTCGTCATCCTTGTAATCA TTAAGAATTTTAGCCATCATAT
0864-pyj335-EcoRV-F	GAGGATATCATGCAAATTAGACAAATACAT
0864-pyj335-KPNI-flag-R	GAGGGTACCTCACTTATCGTCGTCATCCTTGTAATCT ATTTTTTCAAAAGCAGATGTGT
1051-pyj335-EcoRV-F	GAGGATATCATGTTTACAAAAGTGATTGATCC
1051-pyj335-KPNI-flag-R	GAGGGTACCTTACTTATCGTCGTCATCCTTGTAATCA TTAAGCGAGGTCAACTTTTTTGTC
1173-pyj335-EcoRV-F	GAGGATATCATGAGTGAAATCAAACGTCTTG
1173-pyj335-KPNI-flag-R	GAGGGTACCTTACTTATCGTCGTCATCCTTGTAATCT GGTTTCCACAATAAGACATC
2573-pyj335-EcoRV-F	GAGGATATCATGCACCTTTGTCTTCGTTTAAG
2573-pyj335-KPNI-flag-R	GAGGGTACCTTACTTATCGTCGTCATCCTTGTAATCA CCAAGATACACATCTTGATATG
**Oligonucleotides for inverse PCR-based site-directed mutagenesis**	
K63R-F	AGAGTATTTTATAGTTTTATCGATG
K63R-R	TTTGTGCATGCCATTTTCTTTATC
K84R-F	AGAGGTACCATTGCTGGTCAGTATA
K84R-R	CGTTCTGATGACTAATATTTTT
K189R-F	AGAAAAGTAGGTTGGAAAGTTATATTTA
K189R-R	ATCAGTTGGACTTTCTAAAATTG
K266R-F	AGACAACAAACAAATATAGATGTA
K266R-R	GGATGCTTTTCTATCCATAGTAAT
**Oligonucleotides for protein expression**	
0543-pet28a-BamhI-F	GAGGGATCCATGCAAATATATTTAAGTACTT
0543-pet28a-EcoRI-R	GAGGAATTCTTAATAAAAATGTTCTGGAAATTTTA
1173-pet28a-BamhI-F	GAGGGATCCATGAGTGAAATCAAACGTCTTG
1173-pet28a-EcoRI-R	GAGGAATTCTTATGGTTTCCACAATAAGACATC
2573-pet28a-BamhI-F	GAGGGATCCATGCACCTTTGTCTTCGTTTAAG
2573-pet28a-EcoRI-R	GAGGAATTCTTAACCAAGATACACATCTTGATATG
**Oligonucleotides for qRT- PCR**	

hla-F	CAATCAAACCGCCAATTTTT
hla-R	CCTGGCCTTCAGCATTTAAG
gyrB-F	CAAATGATCACAGCATTTGGTACAG
gyrB-R	CGGCATCAGTCATAATGACGAT
0543-F	CAAGCACGTGCGAAAGTAAA
0543-R	TTTTTGTCCACGCAATTCAG
1173-F	GGCATATTTGTCGGAGATCAA
1173-R	CTGCCAATGGCTTTAATTGG
2573-F	GAACTCGGTGGTCAAGGTGT
2573-R	TTTGGCAAATGAACATGAGG

Supplementary Table 3. Virulence factors and their lactylation position

	**Lactylated protein**	**Position**
**Regulatory system**	AgrA	11, 146, 223, 225
	SarA	49, 63, 69, 72, 82
	SarR	52, 76
	MgrA	29, 83, 115, 128, 136, 145
	CodY	16, 18, 47,158, 164, 223, 238, 248, 255
	SrrA	19, 204, 234
**Cytotoxins**	Alpha-toxin	63, 72, 84, 189, 266
	Gamma-toxin	HlgA: 44, 93, 128
	LukAB	F subunit: 275, 330, 335<br>S subunit:45, 59, 110, 124, 149, 158, 165, 182, 232, 286, 303, 315, 340, 346, 349
	EsxA	5, 14, 61, 64
**Adhesion and biofilm formation**	Sbi	329, 339, 344, 349, 377, 412
	Atl	209, 216, 233, 236, 462, 469, 475, 522, 554, 585, 593, 634, 638, 644, 720, 723, 756, 757, 763, 773, 787, 817, 853, 860, 930, 1006, 1089, 1138, 1169, 1229, 1234
	Eap	37, 72, 79, 86, 107, 147, 189, 196, 200, 223, 257, 310, 325, 359, 365, 408, 415, 430, 443, 464, 470, 503, 513, 531, 546, 553, 569, 575, 582, 586, 593, 627, 640, 656, 661, 666, 676, 682
**Protease**	ClpP	164, 167
	FtsH	621, 641
	ClpL	30, 105, 273, 278, 346, 348, 357, 412, 591, 592
	ClpC	326, 451, 514, 716

Supplementary references

1 Kreiswirth, B. N. *et al.* The toxic shock syndrome exotoxin structural gene is not detectably transmitted by a prophage. *Nature* **305**, 709-712 (1983). https://doi.org/10.1038/305709a0

2 Lu, Y. *et al.* Modulation of MRSA virulence gene expression by the wall teichoic acid enzyme TarO. *Nat Commun* **14**, 1594 (2023). https://doi.org/10.1038/s41467-023-37310-5

3 Bae, T. & Schneewind, O. Allelic replacement in Staphylococcus aureus with inducible counter-selection. *Plasmid* **55**, 58-63 (2006). https://doi.org/10.1016/j.plasmid.2005.05.005

4 Liu, Q. *et al.* The ATP-Dependent Protease ClpP Inhibits Biofilm Formation by Regulating Agr and Cell Wall Hydrolase Sle1 in Staphylococcus aureus. *Front Cell Infect Microbiol* **7**, 181 (2017). https://doi.org/10.3389/fcimb.2017.00181

5 Ji, Y., Marra, A., Rosenberg, M. & Woodnutt, G. Regulated antisense RNA eliminates alpha-toxin virulence in Staphylococcus aureus infection. *J Bacteriol* **181**, 6585-6590 (1999). https://doi.org/10.1128/jb.181.21.6585-6590.1999
