# Supplementary material for: Post-translational toxin modification by lactate controls Staphylococcus aureus virulence
Source: Nat Commun. 2024 Nov 13;15:9835. doi: 10.1038/s41467-024-53979-8 (PMC11561239; doi:10.1038/s41467-024-53979-8)
Supplement: Supplementary file 2 — Description of Additional Supplementary Files [file 41467_2024_53979_MOESM2_ESM.pdf]

Description of Additional Supplementary Files:

Supplementary Data 1: Detailed source data for the in vitro lactylase activity assay
