# Supplementary material for: Post-translational toxin modification by lactate controls Staphylococcus aureus virulence
Source: Nat Commun. 2024 Nov 13;15:9835. doi: 10.1038/s41467-024-53979-8 (PMC11561239; doi:10.1038/s41467-024-53979-8)
Supplement: Supplementary file 4 — Reporting Summary [file 41467_2024_53979_MOESM4_ESM.pdf]

nature portfolio

Corresponding author(s): Michael Otto, Min Li
Last updated by author(s): Oct 9, 2024

Reporting Summary

Nature Portfolio wishes to improve the reproducibility of the work that we publish. This form provides structure for consistency and transparency in reporting. For further information on Nature Portfolio policies, see our Editorial Policies and the Editorial Policy Checklist.

Statistics

For all statistical analyses, confirm that the following items are present in the figure legend, table legend, main text, or Methods section.

n/a	Confirmed	
☐	☒	The exact sample size (n) for each experimental group/condition, given as a discrete number and unit of measurement
☐	☒	A statement on whether measurements were taken from distinct samples or whether the same sample was measured repeatedly
☐	☒	The statistical test(s) used AND whether they are one- or two-sided<br>*Only common tests should be described solely by name; describe more complex techniques in the Methods section.*
☐	☒	A description of all covariates tested
☐	☒	A description of any assumptions or corrections, such as tests of normality and adjustment for multiple comparisons
☐	☒	A full description of the statistical parameters including central tendency (e.g. means) or other basic estimates (e.g. regression coefficient) AND variation (e.g. standard deviation) or associated estimates of uncertainty (e.g. confidence intervals)
☐	☒	For null hypothesis testing, the test statistic (e.g. F, t, r) with confidence intervals, effect sizes, degrees of freedom and P value noted<br>*Give P values as exact values whenever suitable.*
☒	☐	For Bayesian analysis, information on the choice of priors and Markov chain Monte Carlo settings
☒	☐	For hierarchical and complex designs, identification of the appropriate level for tests and full reporting of outcomes
☒	☐	Estimates of effect sizes (e.g. Cohen's d, Pearson's r), indicating how they were calculated

Our web collection on statistics for biologists contains articles on many of the points above.

Software and code

Policy information about availability of computer code

Data collection	LC-MS/MS analysis of protein lactylation in S. aureus using the NanoElute UHPLC system (Bruker Daltonics) coupled to a timsTOF Pro mass spectrometer (Bruker Daltonics).<br>The peptide reaction products produced by the activity of lactylases were analyzed by NanoElute UHPLC system (Bruker Daltonics) coupled to a timsTOF Pro2 mass spectrometer (Bruker Daltonics).<br>q-RT-PCR data were obtained on an ABI 7500 thermocycler (Applied Biosystems).<br>The chemiluminescence of immunoblotting analysis was detected on a Tanon 4200 (Tanon).<br>The absorbance was detected using the Synergy2 microplate reader (BioTek).<br>To quantify leukocyte infiltration in the lungs of infected mice, BALF samples were detected by flow cytometry (NovoCyte Advanteon Dx, Agilent).<br>CellSens imaging software (version 4.1) was used to capture tissue paraffin sections for H&E and Ly6G staining (Olympus).
Data analysis	Mass spectrometry data were analyzed using Maxquant (version1.6.15.0). The reference database was S. aureus ST398 (Genome assembly ASM958v1). The Kyoto Encyclopedia of Genes and Genomes (KEGG) database (https://www.kegg.jp/) was used to identify enriched pathways. For each protein category, the InterPro database (https://www.ebi.ac.uk/interpro/) was searched. Gene Ontology (GO) annotation of the proteome was derived from the UniProt-GOA database (http://www.ebi.ac.uk/GOA/). If proteins were not annotated by UniProt-GOA database, the InterProScan soft was used to annotate a protein's GO function based on protein sequence alignment.<br>In lactylase activity assay, the mass spectral data were searched online using PEAKS ONLINE (X Build, version 1.4.2020-10-02_113407). The reference database was the sequence of S. aureus alpha-toxin (GenBank: CAQ49583.1).<br>Fold changes in qRT-PCR experiments and absorbance data were analyzed in Excel (Microsoft Office 2021), and statistical analysis was performed using Graph-Pad Prism for Macintosh (version 9.3.1).

To quantify leukocyte infiltration in the lungs of infected mice, BALF samples were analyzed using NovoExpress software (version 1.5.8). The number/area and proportion of Ly6G positive cells at the lesion were analyzed by using Image J (version 1.51j / Java 1.8.0_112 64-bit).

For manuscripts utilizing custom algorithms or software that are central to the research but not yet described in published literature, software must be made available to editors and reviewers. We strongly encourage code deposition in a community repository (e.g. GitHub). See the Nature Portfolio guidelines for submitting code & software for further information.

Data

Policy information about availability of data

All manuscripts must include a data availability statement. This statement should provide the following information, where applicable:

- Accession codes, unique identifiers, or web links for publicly available datasets
- A description of any restrictions on data availability
- For clinical datasets or third party data, please ensure that the statement adheres to our policy

The MS proteomics data generated in this study can be viewed in PRIDE database (http://www.ebi.ac.uk/pride) by pasting the accession (PXD046022, PXD046023) into the search box. All other data are presented in this manuscript. Source data files (see Supporting Material) contain results for all figures with quantitative data.

Research involving human participants, their data, or biological material

Policy information about studies with human participants or human data. See also policy information about sex, gender (identity/presentation), and sexual orientation and race, ethnicity and racism.

Reporting on sex and gender	Not Applicable
Reporting on race, ethnicity, or other socially relevant groupings	Not Applicable
Population characteristics	Not Applicable
Recruitment	Participants were instructed through notes in the medical center of the hospital. This study used Staphylococcus aureus isolated from patient specimens for molecular mechanism research, without involving patient personal information or disease information.
Ethics oversight	Staphylococcus aureus strains were isolated from patients. All participants or their legal guardians have provided written informed consent to take part in the study. This study was approved by the ethics committee of Renji Hospital, School of Medicine, Shanghai Jiao Tong University, Shanghai (KY2023-060-C).

Note that full information on the approval of the study protocol must also be provided in the manuscript.

Field-specific reporting

Please select the one below that is the best fit for your research. If you are not sure, read the appropriate sections before making your selection.

[x] Life sciences [ ] Behavioural & social sciences [ ] Ecological, evolutionary & environmental sciences

For a reference copy of the document with all sections, see nature.com/documents/nr-reporting-summary-flat.pdf

Life sciences study design

All studies must disclose on these points even when the disclosure is negative.

Sample size	No statistical methods were used to pre-determine sample sizes but our sample sizes were chosen based on those reported in previous publications.<br>For animal studies, our sample sizes were chosen based on those reported data in previous publications and previous experience in similar experimental setups.<br>Ref: Nguyen TH. et al. Nat Microbiol. 2022 Jan;7(1):62-72.<br>Cohen TS. et al. Sci Transl Med. 2016 Mar 9;8(329):329ra31.<br>For LC-MS/MS analysis, sample sizes were chosen that are similar to those used in previous similar studies:<br>Ref: Dong H. et al. Nat Commun. 2022 Nov 4;13(1):6628.<br>Li Z. et al. Sci Signal. 2023 Sep 5;16(801):eadg1849.<br>In vitro experiments were performed with at least three replicates deemed sufficient to achieve reliable results, these experiments have been validated multiple times and similar results have been obtained.
Data exclusions	Data were only excluded in mass spectrometry analysis: the false discovery rate (FDR) was adjusted to < 1% and any identified protein had to contain at least one unique peptide.
Replication	All experiments except LC-MS/MS analysis were repeated and showed similar results.

The LC-MS/MS analysis was not repeated, as it was performed with three replicates deemed sufficient to achieve reliable results.

Randomization	Mice used in the experiments were litter mates and age-matched, and randomized into control and experimental groups. Randomization is not applicable to other (in vitro) experiments.
Blinding	Study did not include any interventions and thus the conventional blinding (as used in clinical trials or intervention studies was not appropriate for this study. For animal experiments, mouse tissue paraffin sections were used for H&E and Ly6G staining. Slides were examined independently by a histopathologist who was blinded to the treatment. Blinding is not applicable to other experiments, but all efforts were made to minimize potential biases through standardized procedures and objective measurement criteria.

Reporting for specific materials, systems and methods

We require information from authors about some types of materials, experimental systems and methods used in many studies. Here, indicate whether each material, system or method listed is relevant to your study. If you are not sure if a list item applies to your research, read the appropriate section before selecting a response.

Materials & experimental systems

n/a	Involved in the study	
☐	☒	Antibodies
☐	☒	Eukaryotic cell lines
☒	☐	Palaeontology and archaeology
☐	☒	Animals and other organisms
☒	☐	Clinical data
☒	☐	Dual use research of concern
☒	☐	Plants

Methods

n/a	Involved in the study	
☒	☐	ChIP-seq
☐	☒	Flow cytometry
☒	☐	MRI-based neuroimaging

Antibodies

Antibodies used

Primary antibodies:
Anti-alpha-hemolysin antibody (Mouse monoclonal antibody, Abcam, Catalog: ab190467, Clone: 8B7) was used to determine expression of alpha-toxin at a dilution of 1:1000.
Anti-L-Lactyl Lysine antibody (Rabbit monoclonal antibody, PTM Biolabs, Catalog: PTM-1401RM) was used to determine lactylation of proteins/peptides at a dilution of 1:1000.
Anti SrtA antibody (Rabbit polyclonal antibody, produced by GLbiochem) was used to determine expression of SrtA at a dilution of 1:1000.
Anti-HlaK84la antibody (Rabbit polyclonal antibody, produced by PTM Biolabs against peptide KLH-VIRTKlaGTIAGRC) was used to determine lactylation of lysine84 of alpha-toxin at a dilution of 1:1000.
Anti-Hla* antibody (Rabbit polyclonal antibody, produced by PTM Biolabs against unmodified peptide VIRTKGTIAGRC coupled to KLH) was used to determine the K84-surrounding region of alpha-toxin at a dilution of 1:1000.
Anti-ADAM10 antibody (Rabbit polyclonal antibody, ABclonal, Catalog: A25167) was used to determine expression of ADAM-10 at a dilution of 1:1000.
Anti-Caveolin-1 antibody (Rabbit monoclonal antibody, ABclonal, Catalog: A19006, Clone: ARC50848) was used to determine expression of Caveolin-1 at a dilution of 1:1000.
Anti-Acetyllysine antibody (Rabbit monoclonal antibody, PTM Biolabs, Catalog: PTM-105RM) was used to determine acetylation of proteins/peptides at a dilution of 1:1000.
PerCP rat anti-mouse CD45 (BD Biosciences, Catalog: 557235, Clone:30-F11) was used to determine expression of CD45 at a dilution of 1:100.
PE Rat Anti-Mouse Ly-6G (BD Biosciences, Catalog: 551461, Clone:1A8) was used to determine expression of Ly-6G at a dilution of 1:100.
Purified anti-mouse CD16/32 Antibody (Mouse monoclonal antibody, BioLegend, Catalog:101302, Clone:93) was used for Fc Block at a dilution of 1:500.
FITC anti-mouse/human CD11b Antibody (BioLegend, Catalog:101206, Clone:M1/70) was used to determine expression of CD11b at a dilution of 1:100.
APC anti-mouse F4/80 Antibody (BioLegend, Catalog:123116, Clone:BM8) was used to determine expression of F4/80 at a dilution of 1:100.
Anti-Ly6G antibody (Rabbit monoclonal antibody, Abcam, Catalog: ab238132, Clone: EPR22909-135) was used to stain Neutrophils at a dilution of 1:1000.

Secondary antibodies:
Anti-rabbit IgG, HRP-linked Antibody, Cell signaling technology, Catalog:7074, Antibody Dilution: 1:5000.
Anti-mouse IgG, HRP-linked Antibody, Cell signaling technology, Catalog:7076, Antibody Dilution: 1:5000.

Validation

Primary antibodies:
Anti-alpha-hemolysin antibody (Mouse monoclonal antibody, Abcam, Catalog: ab190467, Clone: 8B7).
https://www.abcam.com/products/primary-antibodies/alpha-hemolysin-antibody-8b7-n-terminal-ab190467.html

Anti-L-Lactyl Lysine antibody (Rabbit monoclonal antibody, PTM Biolabs, Catalog: PTM-1401RM).
http://www.ptm-biolab.com.cn/productDetail.html?id=5863
Anti-ADAM10 antibody (Rabbit polyclonal antibody, ABclonal, Catalog: A25167)
https://abclonal.com.cn/catalog/A25167
Anti-Caveolin-1 antibody (Rabbit monoclonal antibody, ABclonal, Catalog: A19006, Clone: ARC50848).
https://abclonal.com.cn/catalog/A19006
Anti-Acetyllysine antibody (Rabbit monoclonal antibody, PTM Biolabs, Catalog: PTM-105RM).
http://www.ptm-biolab.com.cn/productDetail.html?id=6244
PerCP rat anti-mouse CD45 (BD Biosciences, Catalog: 557235, Clone:30-F11)
https://www.bdbiosciences.com/zh-cn/products/reagents/flow-cytometry-reagents/research-reagents/single-color-antibodies-ruo/percp-rat-anti-mouse-cd45.557235
PE Rat Anti-Mouse Ly-6G (BD Biosciences, Catalog: 551461, Clone:1A8)
https://www.bdbiosciences.com/zh-cn/products/reagents/flow-cytometry-reagents/research-reagents/single-color-antibodies-ruo/pe-rat-anti-mouse-ly-6g.551461
Purified anti-mouse CD16/32 Antibody (Mouse monoclonal antibody, BioLegend, Catalog:101302, Clone:93)
https://www.biolegend.com/en-us/products/purified-anti-mouse-cd16-32-antibody-190
FITC anti-mouse/human CD11b Antibody (BioLegend, Catalog:101206, Clone:M1/70)
https://www.biolegend.com/en-us/products/fitc-anti-mouse-human-cd11b-antibody-347
APC anti-mouse F4/80 Antibody (BioLegend, Catalog:123116, Clone:BM8)
https://www.biolegend.com/en-us/products/apc-anti-mouse-f4-80-antibody-4071
Anti-Ly6G antibody (Rabbit monoclonal antibody, Abcam, Catalog: ab238132, Clone: EPR22909-135)
https://www.abcam.cn/products/primary-antibodies/ly6g-antibody-epr22909-135-ab238132.html
Validation of anti-Hla* and anti-HlaK84la is included in the manuscript.

Secondary antibodies:
Anti-rabbit IgG, HRP-linked Antibody, Cell signaling technology, Catalog:7074.
https://www.cellsignal.com/products/secondary-antibodies/anti-rabbit-igg-hrp-linked-antibody/7074
Anti-mouse IgG, HRP-linked Antibody, Cell signaling technology, Catalog:7076.
https://www.cellsignal.com/products/secondary-antibodies/anti-mouse-igg-hrp-linked-antibody/7076

Eukaryotic cell lines

Policy information about cell lines and Sex and Gender in Research

Cell line source(s)	A549 cells (Catalog: SCSP-503) were obtained from the Cell Bank of Shanghai Institutes of Biological Sciences, Chinese Academy of Sciences.
Authentication	The A549 cell line is commercial and cell authentication is provided by the cell bank of Shanghai Institute of Biological Sciences. The A549 cell line was authenticated by short tandem repeat (STR) profiling. Amelogenin: X,Y; CSF1PO: 10,12; D13S317: 11; D16S539: 11,12; D5S818: 11; D7S820: 8,11; THO1: 8,9.3; TPOX: 8,11; vWA: 14.
Mycoplasma contamination	No mycoplasma contamination.
Commonly misidentified lines (See ICLAC register)	None of the cell lines used in this study are commonly misidentified as per ICLAC.

Animals and other research organisms

Policy information about studies involving animals; ARRIVE guidelines recommended for reporting animal research, and Sex and Gender in Research

Laboratory animals	BALB/c female mice (6-8 weeks) were purchased from Shanghai JSJ Laboratory Animal Co, Ltd. Animals were housed at 19-26 degrees centigrade at a humidity of 40-70% and with a light/dark cycle of 12h/12h in-house under SPF conditions.
Wild animals	Study did not involve wild animals.
Reporting on sex	Female mice aged between 6-8 weeks were used for experiments. Age-and littermate-matched mice were randomly assigned into treatment groups in each experiment. Staphylococcus aureus is a widely spread pathogen. This study constructed mouse acute infection models to compare the pathogenic differences between wild-type Staphylococcus aureus and mutant strains. Our study does not involve the effect of sex hormones on Staphylococcus aureus infection, and animal gender does not affect the experimental results. Female mice were chosen because they were relatively gentle and less prone to fighting and damaging infected lesions.<br>Mouse models in this study was based on those reported data in previous publications:<br>Malachowa, N. et al. Methods Mol Biol. 2019;1960:139-147.<br>Kim, HK, et al. J Immunol Methods. 2014 Aug;410:88-99.
Field-collected samples	Study did not involve field-collected samples.

Ethics oversight	All animal experiments were performed in accordance with the laboratory animal care and use guidelines of the Chinese Association for Laboratory Animal Sciences (CALAS). Approval was obtained from the Ethics Committee for Experimental Animal Welfare at Renji Hospital, School of Medicine, Shanghai Jiao Tong University, Shanghai (RJ2023-087A).

Note that full information on the approval of the study protocol must also be provided in the manuscript.

Plants

Seed stocks	Not Applicable
Novel plant genotypes	Not Applicable
Authentication	Not Applicable

Flow Cytometry

Plots

Confirm that:

- [x] The axis labels state the marker and fluorochrome used (e.g. CD4-FITC).
- [x] The axis scales are clearly visible. Include numbers along axes only for bottom left plot of group (a 'group' is an analysis of identical markers).
- [x] All plots are contour plots with outliers or pseudocolor plots.
- [x] A numerical value for number of cells or percentage (with statistics) is provided.

Methodology

Sample preparation	To quantify leukocyte infiltration in the lungs of infected mice, the bronchoalveolar lavage fluid (BALF) was collected and centrifuged (400 × g) for five minutes and cells were resuspended in 100 µl of sterile PBS. Cells were incubated with Fc Block.
Instrument	Flow cytometry (NovoCyte Advanteon Dx, Agilent)
Software	NovoExpress software (version 1.5.8)
Cell population abundance	In the lung and BALF, the central immune effector cell is the macrophage. S. aureus induced airway inflammation is usually evaluated by measuring neutrophil infiltration and cytokine expression levels in BALF and lung tissue, which can reflect the virulence of S. aureus and the severity of pneumonia. BALF of mice was collected by lavaging lungs with PBS, neutrophils were defined as CD45+CD11b+ Ly6G+ cells, and macrophages were defined as CD45+CD11b+ F4/80+ cells. In normal mice BALF samples, macrophages were the predominant cell type.
Gating strategy	BALF cells were examined initially by forward scatter (FSC) height versus side scatter (SSC) height, and FSC height versus FSC area, with gating on single cells to eliminate debris and clumped cells from the analysis.<br>Subsequently, a Live/Dead dye was used to eliminate dead cells.<br>Live cells were then examined by CD45 expression, gating on CD45+ cells, which represented total leukocytes.<br>Live CD45+ cells were then examined based on CD11b expression, gating on CD11b+ cells, which represented myeloid cells.<br>Then, neutrophils were defined as CD45+CD11b+ Ly6G+ cells, and macrophages were defined as CD45+CD11b+ F4/80+ cells.<br>Examination of these CD11b+ cells by Ly6G versus F4/80 expression allows the discrimination of two cell populations: Ly6G+ neutrophils and F4/80+ macrophages.

- [x] Tick this box to confirm that a figure exemplifying the gating strategy is provided in the Supplementary Information.
